# The Genetic Identification of Numerous Apicomplexan *Sarcocystis* Species in Intestines of Common Buzzard (*Buteo buteo*)

**DOI:** 10.3390/ani14162391

**Published:** 2024-08-18

**Authors:** Tautvilė Šukytė, Evelina Juozaitytė-Ngugu, Saulius Švažas, Dalius Butkauskas, Petras Prakas

**Affiliations:** Nature Research Centre, Akademijos 2, 08412 Vilnius, Lithuania; tautvile.sukyte@gamtc.lt (T.Š.); evelina.ngugu@gamtc.lt (E.J.-N.); saulius.svazas@gamtc.lt (S.Š.); dalius.butkauskas@gamtc.lt (D.B.)

**Keywords:** *Sarcocystis*, Apicomplexa, Common Buzzard, life cycle, molecular identification, prevalence, phylogeny, *28S rRNA*, *ITS1*

## Abstract

**Simple Summary:**

*Sarcocystis* parasites (Apicomplexa: Sarcocystidae) exhibit a complex life cycle necessitating two hosts and infect a wide range of mammals, birds, and reptiles. Birds of prey, positioned at the apex of the food chain, serve as definitive hosts for various *Sarcocystis* species. This study focuses on the molecular identification of *Sarcocystis* species in the intestines of the Common Buzzard (*Buteo buteo*). For this purpose, the small intestines of 30 birds were tested. Microscopic examination of intestinal scrapings and molecular analysis showed that 73.3% of the birds examined were infected with *Sarcocystis* spp. Nine *Sarcocystis* species using birds or rodents as their intermediate hosts were confirmed in the small intestines of Common Buzzards by means of DNA analysis. The highest detection rates were established for *S. glareoli* (53.3%) forming cysts in the brains of rodents and for *S. halieti* (36.7%) forming sarcocysts in the muscles and brains of birds. In conclusion, the Common Buzzard can transmit numerous *Sarcocystis* spp., including pathogenic ones.

**Abstract:**

The common Buzzard (*Buteo buteo*) was previously shown to transmit two *Sarcocystis* species (*S. glareoli* and *S. microti*) forming cysts in the brains of rodents. Due to a lack of research, the richness of *Sarcocystis* species spread by these birds of prey is expected to be much higher. A total of 30 samples of the small intestine of the Common Buzzard were collected in Lithuania and subjected to *Sarcocystis* species identification based on nested PCR of *28S* rRNA and *ITS1*, following the sequencing of amplified DNA fragments. Six known *Sarcocystis* spp., *S. cornixi*, *S. glareoli*, *S. halieti*, *S. kutkienae*, *S. turdusi*, and *S. wobeseri*, along with three genetically distinct species (*Sarcocystis* sp. Rod3, *Sarcocystis* sp. Rod4, and *Sarcocystis* sp. Rod5), were identified. Phylogenetically, these three potentially new species clustered with *Sarcocystis* spp. characterised by a rodents-birds life cycle. *Sarcocystis* spp. employing rodents and birds as their intermediate hosts were detected in 66.7% and 50.0% of samples, respectively. These findings are consistent with the diet preferences of Common Buzzards. Notably, co-infections with two or more species were observed in a half of the samples. Altogether, the obtained results indicate that the Common Buzzard could serve as definitive host of various *Sarcocystis* species.

## 1. Introduction

The Common Buzzard (*Buteo buteo*) is a numerous and widespread raptor species that breeds throughout Eurasia to the south of the tundra region and up to China, Kazakhstan, Iran, and Turkey in the south [1]. According to the IUCN Red List, the total Common Buzzard population size is between 2.1 and 3.7 million mature individuals, and its population is stable [2]. The European population is estimated at about 814,000–1,390,000 pairs (equating to 1,630,000–2,770,000 mature individuals), while the Lithuanian breeding population is estimated at about 6000–12,000 pairs [3]. It is the most common diurnal raptor in Europe and Lithuania [1,4]. Common Buzzards are year-round residents over much of their distribution range, though birds breeding in the northern and eastern parts of their range are strict or partial migrants [5]. In Lithuania, they occur throughout the year, though most local breeders migrate to wintering sites located in southern Europe and northern Africa [6]. Common Buzzards mainly use a wide range of woodland habitats, but they also breed in agricultural and sub-urban habitats [7]. It is a common breeder in all administrative districts of Lithuania, with an average estimated population density of 14.2 pairs/100 km^2^ of forested area and up to 159.4 pairs/100 km^2^ in optimum habitats [8]. 

*Sarcocystis* parasites (Apicomplexa: Sarcocystidae) are characterised by an obligatory two-host life cycle. They can infect mammals (including humans), reptiles, and birds [9]. Birds of prey are at the top of the food chain and serve as definitive hosts (DHs) of numerous *Sarcocystis* species, mainly using birds and rodents as their intermediate hosts (IHs) [10,11,12]. The DH becomes infected by a consumption of tissues containing mature sarcocysts, while the IH acquires infection through food or water contaminated with sporocysts of *Sarcocystis* spp. Sarcocysts most frequently develop in the muscles and central nervous system (CNS) of the IH, whereas sporulation of oocysts occurs in the small intestine of the DH [9,10,13]. Some species of *Sarcocystis* are pathogenic to their IHs [10,13]. The disclosure of DHs of certain species is very important for identifying pathways of parasite spread. These parasites are described in the IH, while the DH of many *Sarcocystis* species is unknown [10].

The Common Buzzard was identified as the DH of two *Sarcocystis* species, *S. glareoli* and *S. microti*. These species form cysts in the CNS of rodents [12,14,15,16,17,18]. There is evidence indicating that rodents infected with these parasites exhibit a higher risk of being caught by predators compared to uninfected individuals [19]. Previously, two genera were known in the subfamily Sarcocystinae: *Sarcocystis* and *Frenkelia*. Between 1998 and 2000, *Frenkelia* have been synonymised with the genus *Sarcocystis*, and the species *F. glareoli* was reclassified as *S. glareoli*, and *F. microti* was renamed *S. microti* [20,21,22]. For more detailed information, please see the following articles: [10,12,23]. In GenBank, the sequences of *S. microti* and *S. glareoli* are assigned to either the genera *Freneklia* (AF009244, AF009245, AF044251, AF044252, AF076863, AF076864, PP350820, and PP350821) or *Sarcocystis* (PP535695–PP535700).

*Sarcocystis microti* induces infection in a wider IH range than *S. glareoli* and is considered to be less IH-specific. Cysts of *S. microti* have been identified in at least ten genera of rodents [10,24,25,26]. In contrast, the bank vole (*Clethrionomys glareolus*) is considered the main IH of *S. glareoli* [10,27,28]. The determination of IHs of *S. glareoli* and *S. mirroti* was mainly established by microscopical methods [10,14,15,16,26,29,30,31,32]. However, very morphologically similar sarcocysts representing different *Sarcocystis* species are found in closely related IHs. Therefore, DNA analysis methods should be used to confirm the *Sarcocystis* species [12]. The infection of *S. glareoli* has been reported only in Europe [12,27,28,29,30,31,32,33,34,35,36,37], while *S. microti* has been detected worldwide in North America [18], Europe [27,28,29,30,31,32,33,34,35,36,37,38], and Japan [39].

Birds are an important part of the diet of Common Buzzards [1,3,40,41,42,43]. Therefore, in the present study, we aimed to clarify whether Common Buzzards can serve as DHs of *Sarcocystis* spp. using birds as their IHs. Notably, three *Sarcocystis* spp., *S. calchasi*, *S. falcatula*, and *S. halieti*, cause diseases in birds of several different orders, and *S. calchasi* and *S. falcatula* infections can result in mortality of wild birds [44,45,46]. Of these species, *S. calchasi* and *S. halieti* are known to be transmitted via birds of prey [11,47,48,49,50]. Furthermore, eight more species of *Sarcocystis* forming sarcocysts in the tissues of birds are using birds as their DH [10,11,50,51]. Specifically, *S. accipitris*, *S. alectoributeonis*, *S. columbae*, *S. cornixi*, *S. kutkienae*, *S. lari*, *S. turdusi*, and *S. wobeseri* have been shown to be spread by birds of the families Accipitridae and Corvidae [10,11,33,48,49,50,51].

The determination of the DHs of *Sarcocystis* species is complicated. Morphometric measurements of oocysts and sporocysts of different *Sarcocystis* spp. often overlap. Thus, the morphological species identification in DHs is usually impossible [10,11,48,49,50,51]. In addition, it is generally not possible to determine morphologically how many different *Sarcocystis* species are present in a naturally infected predator. It has been repeatedly demonstrated that DHs can be simultaneously infected with sporocysts by several *Sarcocystis* spp. [10,11,48,49,50,51,52,53]. The DH of *Sarcocystis* spp. is often discovered using experimental infection [10,54,55,56]. However, due to ethical issues, such studies are becoming less common in birds of prey and wild predatory mammals [57]. In contrast, molecular methods are increasingly being used to identify *Sarcocystis* species in the small intestine scrapings of naturally infected predators and scavengers [11,12,49,50,51,52,53,58]. Furthermore, phylogenetic analysis can indicate a group of animals (e.g., birds, mammals, and reptiles) as potential DHs of specific *Sarcocystis* species [59,60,61,62]. For instance, *Sarcocystis* species using birds as their IH are placed into two clusters based on parasite DHs, birds of prey, or predatory mammals [59].

The mitochondrially encoded cytochrome c oxidase I (*cox1*) is the best choice for the genetic identification of *Sarcocystis* species that use ungulates as IHs [63,64]. However, this locus is too conservative to discriminate *Sarcocystis* spp. forming sarcocysts in muscles or CNS of birds and rodents. The *ITS1* (internal transcribed spacer 1) is the most reliable marker for identifying *Sarcocystis* spp. using birds as IHs [61,65]. The *28S* rRNA was shown to be a useful gene for the detection of *Sarcocystis* spp. that use rodents as their IH [12,36,37]. 

In a previous study, we identified *S. glareoli* in 5 of 10 examined intestinal mucosa scraping samples of the Common Buzzard collected in Lithuania [12]. However, in accordance with the diet of these birds, we assumed that the *Sarcocystis* species richness spread by the Common Buzzard is considerably higher. Therefore, we continued our research to determine the role of the Common Buzzard in transmitting *Sarcocystis* spp. The objective of the present study was, by means of DNA analysis, to identify *Sarcocystis* spp. using birds and rodents as their IHs in the intestines of the Common Buzzard. 

## 2. Materials and Methods

### 2.1. Collection of Common Buzzard Samples

A total of 30 Common Buzzards (isolates BbLT11–BbLT40) were collected between 2020 and 2023 in different districts of Lithuania, mainly in Trakai and Ukmergė. All birds obtained from the Kaunas Tadas Ivanauskas Zoology Museum (the Lithuanian national authority responsible for monitoring dead birds) were found dead as a result of collisions with motor vehicles, power lines, buildings, etc., and were kept frozen at −20 °C until microscopic and molecular examinations had been conducted. The current investigation was approved by the Animal Welfare Committee of the Nature Research Centre (no. GGT-9). 

### 2.2. Preparation of Intestines and Microscopical Characterisation of Sarcocystis spp.

*Sarcocystis* spp. were isolated from the intestines of each Common Buzzard employing a modified technique based on Verma et al. [66]. This method is suitable for the isolation of the different developmental stages of *Sarcocystis* spp. including oocysts, sporulated oocysts, and sporocysts. The necropsy of the bird was prepared by making a longitudinal incision in the intestine and scraping the mucosal surface. The exfoliated epithelium was immersed in 50 mL of distilled water (dH2O) and homogenised with a commercial mixer at high speed for 1–2 min. The resulting homogenate was centrifuged at 1600 rpm for 6 min at 20 °C and the supernatant removed. The remaining sediments were resuspended in 50 mL of fresh distilled water. Homogenisation, centrifugation, and washing were repeated until the supernatant remained clear. The resulting sediments was diluted 1:1 with HBSS (Hank’s balanced salt solution) and 5.25% sodium hypochlorite solution. The mixture was incubated in an ice bath for 30 min. The centrifugation procedure was then repeated by partial decantation of the supernatant and addition of distilled water until the odour of sodium hypochlorite disappeared. The obtained sediments were evaluated for oocysts/sporocysts under a light microscope (LM) at ×400 magnification. At the last step, 400 µL of resuspended sediment was used from each sample for DNA isolation. DNA extraction was conducted for all bird samples, irrespective of the presence of *Sarcocystis* spp. oocysts/sporocysts. 

### 2.3. Molecular Analysis of Sarcocystis spp.

The isolation of genomic DNA from intestinal scrapings of Common Buzzards was performed using the GeneJET Genomic DNA Purification Kit (Thermo Fisher Scientific Baltics, Vilnius, Lithuania). 

The identification of *Sarcocystis* spp. employing birds as IHs was carried out using a previously described method based on species-specific nested PCR of *ITS1* and sequencing [11,51]. The primers used for the amplification of *ITS1* fragments are listed in Table 1. In this way, the presence of *S. calchasi*, *S. columbae*, *S. cornixi*, *S. corvusi*, *S. fulicae*, *S. halieti*, *S. kutkienae*, *S. lari*, *S. turdusi*, and *S. wobeseri* was checked in the samples analysed. The following 10 species were previously reported in Lithuania. It is known or suggested by phylogenetic results that these *Sarcocystis* species use birds as their DH. The SU1F/5.8SR2 primer pair was applied in the first round of the nested PCR, while the following ten primer pairs were used to identify target *Sarcocystis* species from birds.

The detection of *Sarcocystis* spp. employing rodents as IHs was performed by nested PCR of *28S* rRNA and *ITS1* followed by sequencing. Some of the primers used for the amplification of these species were designed in the current study by the Primer3Plus program (Table 1). For the design of GsSglajamF1/GsSglajamR1 primer, we used recently isolated DNA of *S. glareoli* isolated from four individual cysts retrieved from the brains of four individual bank voles [36]. To amplify the *28S* rRNA, Sgrau281 and Sgrau282 primers were used in the first step of the nested PCR. In a second round of nested PCR, we used three primer pairs, GsSglaF1/GsSglaR1, GsSmicF1/GsSmicR1 and SgraupaukF/SgraupaukR, theoretically amplifying *S. glareoli*, *S. microti,* and *Sarcocsystis* spp. using rodents as their IHs, respectively. Fragments of the *ITS1* region were amplified using the external primer pair SU1F/5.8SR2 and the internal primers GsSglajamF1 and GsSglajamR1. PCRs were conducted in a 25 μL mixture that included 4 μL of template DNA, 0.5 μM of both forward and reverse primers, 12.5 μL of DreamTaq PCR Master Mix (Thermo Fisher Scientific Baltics, Vilnius, Lithuania), and the remaining amount of nuclease-free water. Three negative controls were used: (i) a control of the first amplification step using nuclease-free water instead of DNA template, (ii) a control of the second step of nested PCR using water instead of the target DNA, and (iii) a control of the second step of nested PCR adding 1 μL of negative control of the first step. The PCR was initiated with an initial hot start at 94 °C for 5 min, followed by 35 cycles of 94 °C for 35 s, annealing for 54 to 61 °C according to the primers for 45 s and 72 °C for 55 s, and a final extension at 72 °C for 5 min. The amplified products were visualised using 1.0% agarose gel electrophoresis and purified with the help of ExoI and FastAP (Thermo Fisher Scientific Baltics, Vilnius, Lithuania). 

The PCR products were sequenced directly using the same forward and reverse primers as for the PCR. Sequencing reactions were performed using the Big-Dye^®^ Terminator v3.1 Cycle Sequencing Kit and the 3500 Genetic Analyzer (Applied Biosystems, Foster City, CA, USA) according to the manufacturer’s recommendations. The resulted sequences were compared with those of various *Sarcocystis* spp. available in NCBI GenBank using the nucleotide BLAST (http://blast.ncbi.nlm.nih.gov/, accessed on 19 June 2024). Notably, only pure DNA sequences without double peaks or polysignals were used for further analysis, whereas impure sequences obtained in several cases indicating sequencing errors or mixed *Sarcocystis* species infections were excluded from further investigations. The *Sarcocystis* spp. sequences generated in the current work were submitted to GenBank with the accession numbers PP937189, PP937483–PP937522, and PP938236–PP938271.

### 2.4. Phylogenetic Analysis

The phylogenetic relationships of the detected *Sarcocystis* spp. using birds as IHs were disclosed in our previous works [11,51,62]. In the current study, we obtained potentially new *Sarcocystis* species using rodents as IHs; therefore, the phylogenetic analysis focused on the relationships between these species. 

Phylogenetic testing was carried out with the help of MEGA11 version 11.0.13 software [67]. Multiple sequence alignments of partial *28S rRNA* and *ITS1* sequences were performed using the MUSCLE algorithm. The reconstruction of phylogenetic relationships was conducted using the Maximum Likelihood (ML) approach. The selection of nucleotide evolution model has been made using the “Find Best DNA/Proteins Models (ML)” option by the generated lowest scores of Bayesian Information Criterion. Thereby, the Tamura 3 parameter + G evolutionary model was proposed to examine the phylogenetic relationships of the *28S* rRNA and *ITS1* sequences. Gaps/missing data were treated using the complete deletion option. *Toxoplasma gondii* and *S. falcatula* were set as outgroups of the *28S* rRNA and *ITS1* analyses, respectively. The reliability of the resulted trees was assessed by the bootstrap method with 1000 replications.

## 3. Results

### 3.1. Microscopical Detection of Sarcocystis spp. Oocysts/Sporocyst

**Figure 1:**
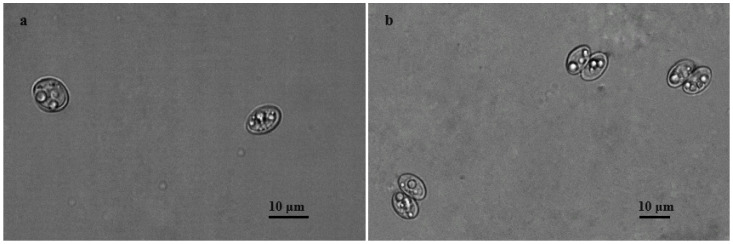
Sporocysts (**a**) and sporulated oocysts (**b**) of *Sarcocystis* spp. found in intestinal scrapings of Common Buzzards.

The examination of the intestinal scraping samples under a light microscope revealed *Sarcocystis* spp. infection in 22 of 30 (73.3%) Common Buzzards. Under LM, the detected sporocysts were 10.8–18.2 × 8.1–13.9 μm (14.6 × 10.9 μm; *n* = 83) in size, while the sporulated oocysts measured 8.1–16.3 × 15.3–21.5 μm (12.8 × 17.4 μm; *n* = 27) (Figure 1).

### 3.2. Molecular Identification of Sarcocystis spp. Using Birds as Their Intermediate Hosts and Common Buzzards as Definitive Hosts

Five different *Sarcocystis* species, *S. cornixi*, *S. halieti*, *S. kutkienae*, *S. turdusi*, and *S. wobeseri*, employing birds as their IHs, have been identified in the intestines of the Common Buzzard (Table 2). Our *ITS1* sequences, excluding primer binding sites, ranged from 435 bp to 596 bp. Eleven obtained sequences of *S. halieti* showed 97.3–100% similarity compared to each other, two sequences of *S. kutkienae* generated in this work differed by 0.5%, while no intraspecific polymorphism was observed while analysing the other species detected. Based on the analysed *ITS1* fragments, the intraspecific genetic similarity values, calculated using sequences obtained in the present study and those available in GenBank, were 96.3–100%. By contrast, the intraspecific genetic similarity values obtained comparing with the most closely related species did not exceed 92.7%. Thus, the calculated intraspecific and interspecific genetic similarity values for all five detected species did not overlap, verifying the accuracy of species identification. 

### 3.3. Molecular Characterisation of S. glareoli and Three Potentially New Sarcocystis Species Detected in Intestines of Common Buzzards

At first, three primer pairs (GsSglaF1/GsSglaR1, GsSmicF1/GsSmicR1, and SgraupaukF/SgraupaukR) amplifying *28S* rRNA fragments (Table 1) were used for the screening of *S. glareoli*, *S. microti*, and other *Sarcocystis* spp. using rodents as their IHs. No amplification was obtained with GsSmicF1/GsSmicR1. Nineteen samples appeared to be positive using GsSglaF1/GsSglaR1 and/or SgraupaukF/ SgraupaukR primer pairs (Table S1). The *28S* rRNA sequences generated in 16 isolates of Common Buzzards showed 99.8–100% similarity to those *S. glareoli*, 99.6–99.7% similarity to those of *S. jamaicensis*, and 99.3–99.6% similarity to those of *Sarcocystis* sp. Rod3. Meanwhile *28S* rRNA sequences obtained in two isolates of Common Buzzards (BbLT18 and BbLT36) demonstrated 100% similarity to those of *Sarcocystis* sp. Rod3., 99.8% similarity to those of *S. jamaicensis*, and 99.4–99.7% similarity to those of *S. glareoli* (Tables S1 and S2).

Based on the analysed *28S* rRNA fragments, minor genetic differences between *S. glareoli, S. jamaicensis*, and *Sarcocystis* sp. Rod3 were observed. Therefore, for the separation of these taxa, we decided to design a primer targeting highly variable *ITS1*. As a result, four 100% identical sequences of *S. glareoli* containing partial *18S* rRNA, complete *ITS1*, and partial *5.8S* rRNA were obtained. The *ITS1* region sequence was submitted to GenBank under accession number PP937189. This 1006 bp sequence displayed 93.0% similarity (98% query coverage) compared to *S. jamaicensis* (KY994651) and less than 85% compared to other *Sarcocystis* spp. available. Afterwards, the GsSglajamF1/GsSglajamR1 primer pair was designed in silico to amplify *S. glareoli* and *S. jamaicensis*. By using GsSglajamF1/GsSglajamR1 primers, we have obtained eighteen 518 bp *ITS1* sequences. Among them, 16 sequences (PP937483–P937498) showed 100% identity to each other and to that of *S. glareoli* (PP937189) and 88.6% similarity to that of *S. jamaicensis* (KY994651) (Table 3). The other two identical sequences (PP937499 and PP937500) demonstrated 98.5% similarity to those of *S. glareoli* (PP937189 and PP937483–P937498) and 87.6% similarity to that of *S. jamaicensis* (KY994651). We obtained these two sequences in the same isolates of Common Buzzards (BbLT18 and BbLT36) in which the generated *28S* rRNA sequences displayed 100% identity to *Sarcocystis* sp. Rod3 (Table S1). Thus, based on *28S* rRNA and *ITS1* sequences, *S. glareoli* and presumably another taxon preliminary named *Sarcocystis* sp. Rod3 were established. At *ITS1*, these two taxa differed by 1.5%, which were due to eight stable single-nucleotide polymorphisms (SNPs).

Furthermore, with the help of the SgraupaukF/SgraupaukR primer pair, we have obtained three *28S* rRNA sequences demonstrating ≤95.0% similarity compared to *S. glareoli*, *S. jamaicensis*, *S. microti,* and *Sarcocystis* sp. Rod3 (Table S2). Among them, two identical sequences (PP938269 and PP938270) showed 97.7% similarity to the third one (PP938271), 100% similarity to that of *Sarcocystis* sp. Rod4 (PP535702), and 96.2–96.6% similarity to those of *S. strixi*, *S*. cf. *strixi*, and *S. funereus* (MF162316, MW349707, OQ557459, OR725602, OR726006, and PP350819). The third *28S* rRNA sequence (PP938271) displayed 97.4–97.7% similarity to those of *S. strixi* and *S*. cf. *strixi* (MF162316 and OQ557459). Thus, the genetic comparison showed that the discussed sequences belong to two taxa, which were preliminary named *Sarcocystis* sp. Rod4 and *Sarcocystis* sp. Rod5 (Table 3). In summary, four different taxa, *S. glareoli*, and three not-yet-described genetically different species (*Sarcocystis* sp. Rod3, *Sarcocystis* sp. Rod4, and *Sarcocystis* sp. Rod5) were identified using primers designed for the diagnosis of *Sarcocystis* spp. using rodents as their IH.

### 3.4. Phylogeny of Sarcocystis spp. Identified in Common Buzzards Using Rodents as Their Intermediate Hosts

**Figure 2:**
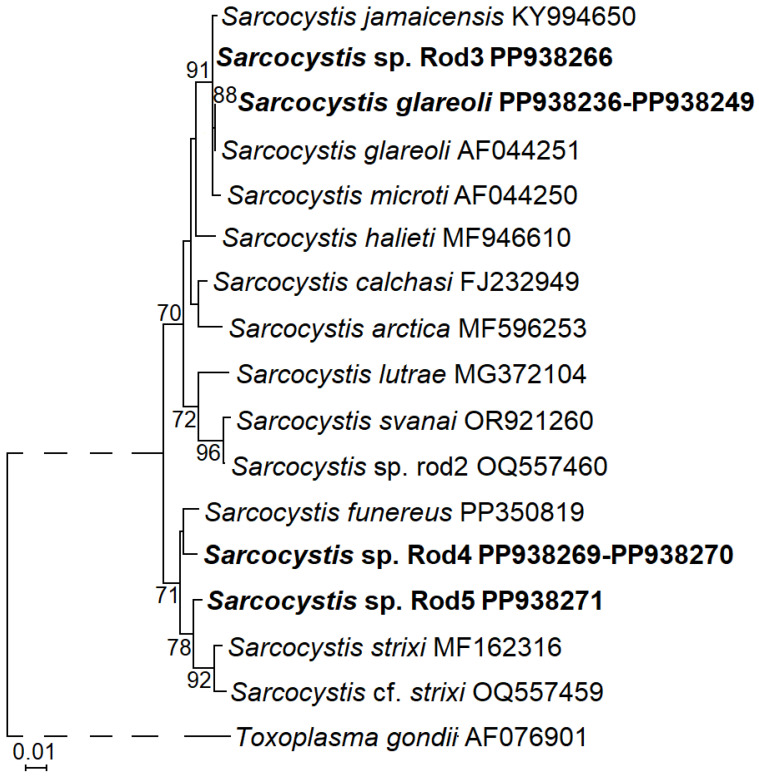
A phylogram of selected *Sarcocystis* species based on *28S* rRNA displaying the phylogenetic placement of four identified species using rodents as their IH and Common Buzzards as their DH. The sequences obtained in our study are in boldface. The ML method and the Tamura 3 parameter + G nucleotide substitution model was used for the generation of the phylogenetic tree. The multiple alignment contained 20 taxa and 678 nucleotide positions including gaps. The phylogram was scaled according to branch length and rooted on *Toxoplasma gondii*. The dashed line does not reflect the actual evolutionary distance between taxa. The figures next to the branches show bootstrap values higher than 70. Sequences obtained in the present study are shown in boldface.

Based on partial *28S* rRNA sequences, *S. glareoli* clustered with *S. microti* and *S. jamaicensis* with a high support (Figure 2). *Buteo* Hawks were proved to be DHs of these three *Sarcocystis* species [12,15,16,17,18,50,68]. However, the analysed fragment appeared to be too conservative for the disclosure of the phylogenetic relationships between the discussed *Sarcocystis* species. Other two species identified, *Sarcocystis* sp. Rod4 and *Sarcocystis* sp. Rod5, were most closely related to *S. funereus*, *S. strixi*, and *Sarcocystis* cf. *strixi*. Specifically, *Sarcocystis* sp. Rod5 clustered with *S. strixi* and *Sarcocystis* cf. *strixi*, while *Sarcocystis* sp. Rod4 was more closely related to *S. funereus* than to *S. strixi* and *Sarcocystis* cf. *strixi*. The literature data show that these species cycle between rodents and birds of prey [36,37,69,70,71]. Thus, the phylogenetic results confirm the identification of four different *Sarcocystis* species using rodents as their IH in the intestines of Common Buzzards. 

**Figure 3:**
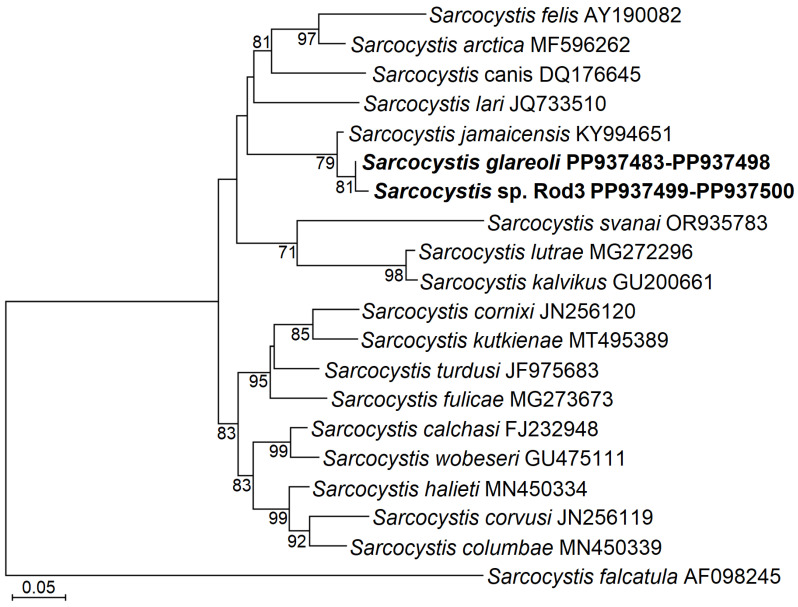
A phylogram of selected *Sarcocystis* species based on *ITS1* showing the phylogenetic position of *S. glareoli* and *Sarcocystis* sp. Rod3 identified in intestines of Common Buzzards. The sequences obtained in our study are marked in bold. The ML method and the Tamura 3 parameter + G nucleotide substitution model was applied for the construction of the phylogenetic tree. The multiple alignment contained 17 taxa and 626 nucleotide positions including gaps. The phylogram was scaled according to branch length and rooted on *S. falcatula*. Bootstrap values higher than 70 are demonstrated next to the branches. Sequences obtained in the present study are shown in boldface.

In the phylogenetic tree based on partial *ITS1* sequences, *S. glareoli* and *Sarcocystis* sp. Rod3 were grouped together, whereas *S. jamaicensis* was a sister species to this cluster (Figure 3). These three species using *Buteo* Hawks as their DH were more closely related to *Sarcocystis* spp. employing predatory mammals and birds as their IH and DH, respectively (e.g., *S. arctica* and *S. lutrae*), than to those employing birds as their hosts (e.g., *S. calchasi* and *S. halieti*). 

### 3.5. The Prevalence of Sarcocystis spp. Identified in the Common Buzzard from Lithuania 

The most frequently identified *Sarcocystis* species was *S. glareoli*, which was found in more than half of samples (53.3%). A relatively high detection rate was also recorded for *S. halieti* (36.7%) and *S. wobeseri* (23.3%). By contrast, other *Sarcocystis* species that were established were rarely found. *Sarcocystis kutkienae*, *Sarcocystis* sp. Rod3, and *Sarcocystis* sp. Rod4 were confirmed in two samples, while *S. cornixi*, *S. turdusi*, and *Sarcocystis* sp. Rod5 were diagnosed in a single sample. 

**Figure 4:**
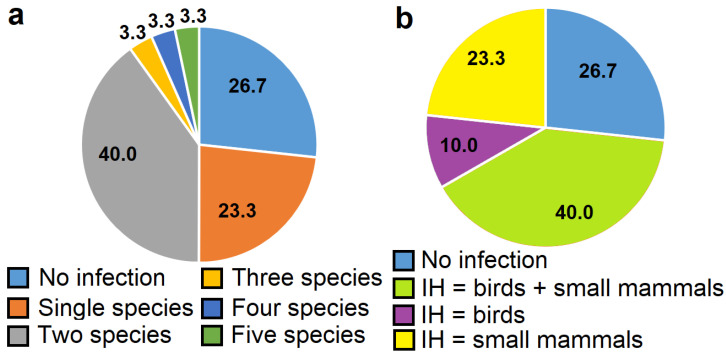
The percentage distribution of *Sarcocystis* spp. in the examined sample of Common Buzzards from Lithuania. (**a**) The distribution of the number of species identified in the sample. (**b**) The distribution of *Sarcocystis* spp. using birds and or rodents as their IH in the sample. IH = intermediate host.

By means of DNA analysis methods, at least one *Sarcocystis* species was identified in 73.3% of samples examined, as it was also confirmed by morphological examination (Figure 4a). The detection of more than one *Sarcocystis* species was determined in the half of samples; two different parasite species were observed in 40.0% of the samples, while three to five parasite species were established in 10.0% of the samples. The highest proportion, i.e., 40.0% of the samples, were positive for *Sarcocystis* spp. employing birds and rodents as their IH (Figure 4b). However, the identification of *Sarcocystis* spp. using rodents as their IH compared to parasite species using birds as their IH was more frequent. Only 10.0% of samples were positive for exclusively *Sarcocystis* spp. using birds as their IH, whereas in 23.3% of samples only *Sarcocystis* spp. using rodents as their IH were established. Thus, *Sarcocystis* spp. employing rodents and birds as their IH were identified in 66.7% and 50.0% of the samples, respectively. No more than four different species of *Sarcocystis* spp. forming sarcocysts in birds and no more than two species of *Sarcocystis* spp. forming sarcocysts in rodents were detected per sample. 

## 4. Discussion

### 4.1. The Role of Common Buzzards in the Transmission of Sarcocystis spp. Using Rodents as Their IH

In the intestines of Common Buzzards, we have identified four different taxa, *S. glareoli*, and three potentially new *Sarcocystis* species (*Sarcocystis* sp. Rod3, *Sarcocystis* sp. Rod4, and *Sarcocystis* sp. Rod5), using rodents as their IH. Notably, the incidence of these *Sarcocystis* species was higher than those employing birds as their IH (Figure 4b). However, even 40.0% of Common Buzzards were positive for *Sarcocystis* species using birds and rodents as their IH. The obtained results can be explained by the dietary preferences of Common Buzzards. These birds are generalist carnivores, and their diet includes rodents, birds, reptiles, amphibians, large insects, and carrion [1,3,40,41,42,43]. Notably, the diet can be different in various regions and habitats as it is a very adaptable species. In Lithuania, the ration of Common Buzzards consists of up to 38.3% of small rodents, primarily voles, and up to 31.9% of small birds [6]. 

In the current study, *S. microti* was not detected in the intestinal scrapings of Common Buzzards. These findings may reflect the low prevalence of *S. microti* in rodents observed in Lithuania, where the infection rates were as low as 2.9% (1/35) in tundra voles (*Alexandromys oeconomus*) and 4.2% (1/24) in short-tailed voles (*Microtus agrestis*) [31]. Furthermore, the most recent study reported no positive results for *S. microti* detection in the muscles of 694 rodents and in the intestines of 10 Common Buzzards in Lithuania [12]. Low infection rates of *S. microti* were also reported in France. Cysts of the parasite were identified in short-tailed voles, common voles (*Microtus arvalis*), and bank voles, with the prevalence varying between 1.0% and 9.2% [27]. Likewise, in the Czech Republic, *S. microti* was found in common vole, bank vole, and *Apodemus* sp., with infection prevalence ranging from 0.6% to 5.0% among the tested species [33].

In the present study, *S. glareoli* was identified in 53.3% of Common Buzzards. This finding corresponds with the high prevalence of *S. glareoli* in the muscles of rodents. For example, bank voles consistently exhibit high infection rates with *S. glareoli* across various regions, indicating a robust host–parasite relationship. The prevalence of *S. glareoli* in bank voles was found to be 21.1% in Lithuania, 47.3% in France, and 55.9% in Germany [27,28,31]. We have for the first time obtained complete *ITS1* sequences of *S. glareoli*. The newly designed primer pair GsSglajamF1/GsSglajamR1 targeting *ITS1* turned out to be very beneficial in discriminating *S. glareoli* from the closely related *S. jamaicensis* [12,68,72]. 

### 4.2. The Role of Common Buzzards in the Transmission of Sarcocystis spp. Using Birds as Their IH

In the present study, for the first time, *S. cornixi*, *S. halieti*, *S. kutkienae*, *S. turdusi*, and *S. wobeseri* using birds as IHs were established in the intestinal samples of Common Buzzards. Corvids are known to be IHs of three *Sarcocystis* species (*S. cornixi*, *S. halieti*, and *S. kutkienae*) identified in the current study. According to current knowledge, *S. cornixi* and *S. kutkienae* form sarcocysts exclusively in the muscles of Corvidae birds [62,73,74], whereas *S. turdusi* forms sarcocysts in the muscles of small passeriform birds of the families Turdidae [75] and Muscicapidae (see KJ540167 GenBank record). Notably, *S. halieti* and *S. wobeseri* are multi-host adapted species, using birds of several different orders as their IH [46,49,74,76,77,78,79,80,81,82,83]. It is worth noting that Accipitridae birds were confirmed to serve as IHs of both these *Sarcocystis* species [11,48,49].

Based on DNA sequence analysis, Northern Goshawk (*Accipiter gentilis*) was shown to be a potential DH of the five discussed *Sarcocystis* species identified in this work, whereas *S. halieti*, *S. turdusi*, and *S. wobeseri* were confirmed in intestinal scrapings of Eurasian Sparrowhawks (*Accipiter nisus*) in Lithuania [11]. Furthermore, all these five *Sarcocystis* species (*S. cornixi*, *S. halieti*, *S. kutkienae*, *S. turdusi*, and *S. wobeseri*) were molecularly detected in the intestines of corvids collected in Lithuania [51]. In this study, of the analysed species, *S. halieti* and *S. wobeseri* were the most frequently identified in the Common Buzzard. IHs of *S. halieti* are birds of prey, small birds, terrestrial birds, and water birds, whereas water birds and birds of prey are known to serve as IHs of *S. wobeseri* [46,49,74,76,77,78,79,80,81,82,83]. Due to the lack of research on the diversity of *Sarcocystis* species in birds, it is assumed that the circle of IHs of *S. wobeseri* is wider [81,82,83]. Thus, the highest detection rates of *S. halieti* and *S. wobeseri* can be explained by their multi-host adaptivity.

In this work, *S. calchasi*, *S. columbae*, *S. corvusi*, and *S. lari* were not found in the intestine samples of Common Buzzards. In Lithuania, highly pathogenic *S. calchasi* was detected only in the intestinal scrapings of 1 out of 16 Northern Goshawks (6.3%), and the assumption was raised that this species is not widespread in Lithuania [11]. *Sarcocystis corvusi* is suspected to be extremely rare in the country [74]. Other species *S. columbae* have been found in the muscles of the Wood Pigeon (*Columba palumbus*) [84], Herring Gull (*Larus argentatus*) [77], Common Gull (*Larus canus*), and Black-Headed Gull (*Larus ridibundus*) [80]. Sarcocysts of *S. lari* were detected in the muscles of the Great Black-Backed Gull (*Larus marinus*) [59] and the Herring Gull [77]. In conclusion, the composition of identified *Sarcocystis* spp. using birds as IHs in the analysed samples is generally consistent with the diet of the Common Buzzard which favours small birds in its diet [1,3,6,40,41,42,43].

### 4.3. Common Buzzards as Definitive Hosts of Potentially New Sarcocystis Species

In our recent investigation, we have identified two potentially new *Sarcocystis* species in the intestinal mucosa scrapings of two tested Rough-Legged Buzzards (*Buteo lagopus*). These species were named *Sarcocystis* sp. Rod3 and *Sarcocystis* sp. Rod4 [12]. Based on *28S* rRNA, *Sarcocystis* sp. Rod3 and *Sarcocystis* sp. Rod4 were shown to be most closely related with *Sarcocystis* spp. using rodents as their IH. The same two species were also detected in the present work. Thus, it could be assumed that Rough-Legged Buzzards and Common Buzzards can potentially spread the same *Sarcocystis* species. Such a finding is not surprising, as *Sarcocystis* species are usually more specific to their IH than to their DH [10]. 

Based on *ITS1*, *Sarcocystis* sp. Rod3 was clearly differentiated from *S. jamaicensis* and was most closely related to *S. glareoli*. At 518 bp long *ITS1* fragment analysed, *Sarcocystis* sp. Rod3, and *S. glareoli* differed by eight SNPs, which represents 98.5% sequence similarity (Table 3 and Table S2). Such a genetic difference in highly variable *ITS1* should be treated as relatively small [82]. However, no variability was observed when comparing four complete *ITS1* sequences of *S. glareoli* isolated from the IH, the bank vole, and 16 partial *ITS1* sequences of *S. glareoli* isolated from the DH, the Common Buzzard. Thus, to clarify whether *Sarcocystis* sp. Rod3 represents a separate species or a distant genetic lineage of *S. glareoli*, detailed morphological studies of this parasite in IHs are required. Furthermore, molecular examinations of small intestine samples of Red-Tailed Hawks (*Buteo jamaicensis*) and Red-Shouldered Hawks (*Buteo lineatus*) in the USA suggest the presence of at least one separate *Sarcocystis* species closely related to *S. glareoli*, *S. jamaicensis*, and *S. microti* [12,50]. 

In the current work on the intestinal mucosa of the single Common Buzzard, we identified *Sarcocystis* sp. Rod5 (Table S1). This potentially new *Sarcocystis* species was found for the first time. *Sarcocystis* sp. Rod5 as well as *Sarcocystis* sp. Rod4 phylogenetically clustered with *S. funereus* and *S. strixi* (Figure 2) using the Tengmalm’s Owl (*Aegolius funereus*) and the Barred Owl (*Strix varia*) as their experimentally confirmed DH. The molecular detection of *Sarcocystis* sp. Rod4 and *Sarcocystis* sp. Rod5 in the intestines of Common Buzzards reinforces our previously proposed assumption that some *Sarcocystis* spp. might be transmitted by representatives of Accipitriformes and Strigiformes [12]. It would therefore be beneficial to further test the intestines of strigiforms for the presence of certain *Sarcocystis* species using accipitriforms as their DH. To summarise, the applied molecular methods allowed us to detect several potentially new *Sarcocystis* species yet not described in IHs. However, cloning of PCR products amplified using primers specific to the whole *Sarcocystis* genus [57,85,86,87,88] can potentially identify yet-not-detected species.

## 5. Conclusions

Based on nested PCR of *28S* rRNA and/or *ITS1* sequences, we have identified nine *Sarcocystis* spp. in the small intestine mucosa of Common Buzzards from Lithuania. Five of these species, *S. cornixi*, *S. halieti*, *S. kutkienae*, *S. turdusi*, and *S. wobeseri*, using birds as their IH, were for the first time established in this bird of prey. In addition, *S. glareoli* forming cysts in the brains of rodents and three yet-undescribed species, *Sarcocystis* sp. Rod3, *Sarcocystis* sp. Rod4, and *Sarcocystis* sp. Rod5, on the basis of phylogenetic results employing rodents as their IH, were detected. The distribution of identified *Sarcocystis* species was in congruence with the diet of Common Buzzards.

Several taxa, such as *S. glareoli*, *S. jamaicensis*, *S. microti*, and *Sarcocystis* sp. Rod3, showed low interspecific variability within *28S* rRNA. Therefore, in the current study, we suggested a primer pair GsSglajamF1/GsSglajamR1 targeting *ITS1* for the clear molecular separation of *S. glareoli* and *S. jamaicensis* on the basis of the resulting DNA sequences. Thus, the *ITS1* might be the appropriate locus for the distinguishment of closely related *Sarcocystis* species using rodents as their IH. Despite the fact that we have identified as many as three potentially new *Sarcocystis* species in this work, further refinement of the methods, for instance, using cloning of PCR products amplified using pan-specific primers, would allow better identification of mixed infections and elucidation of *Sarcocystis* species composition in the intestines of Common Buzzards or other birds of prey.

## Figures and Tables

**Table 1:** List and characteristics of primers used for amplification of various *Sarcocystis* spp.

Primer Name	Orientation	Primer Sequence (5′-3′)	Locus	Length (bp)	Target Species	Ref.
SU1F	Forward	GATTGAGTGTTCCGGTGAATTATT	*ITS1* region *	~1000	*Sarcocystis* spp.	[49]
5.8SR2	Reverse	AAGGTGCCATTTGCGTTCAGAA				
GsScalF2	Forward	CCTTTTGTAAGGTTGGGGACATA	*ITS1*	584	*S. calchasi*	[11]
GsScalR2	Reverse	GCCTCCCTCCCTCTTTTTG				
GsScolF	Forward	ATATGTTCATCCTTTCGTAGCGTTG	*ITS1*	579	*S. columbae*	[51]
GsScolR	Reverse	GCCATCCCTTTTTCTAAGAGAAGTC				
GsScornF2	Forward	AGTTGTTGACGTTCGTGAGGTC	*ITS1*	483	*S. cornixi*	[51]
GsScornR2	Reverse	ACACACTACTCATTATCTCCTACTCCT				
GsScovF	Forward	TATTCATTCTTTCGGTAGTGTTGAG	*ITS1*	524	*S. corvusi*	[51]
GsScovR	Reverse	TTACTCTTTTAACAGCTTCGCTGAG				
GsSfulF	Forward	CAAAGATGAAGAAGGTATATACGTGAA	*ITS1*	449	*S. fulicae*	[51]
GsSfulR	Reverse	CTTTACTCTTGAAGAACGACGTTGA				
GsShalF	Forward	GATAATTGACTTTACGCGCCATTAC	*ITS1*	644	*S. halieti*	[51]
GsShalR2	Reverse	CCATCCCTTTTTCTAAAGGAGGTC				
GsSkutkF2	Forward	ACACACGGTCGAGTTGATATGAC	*ITS1*	625	*S. kutkienae*	[51]
GsSkutkR2	Reverse	TCTTTACCCTTAAACAATTTCGTTG				
GsSlarF	Forward	TTCGTGAGGTTATTATCATTGTGCT	*ITS1*	545	*S. lari*	[51]
GsSlarR	Reverse	GGCGATAGAAATCAAAGCAGTAGTA				
GsSturF	Forward	GATTTTTGATGTCCGTTGAAGTTAT	*ITS1*	561	*S. turdusi*	[51]
GsSturR	Reverse	CATTCAAATATGCTCTCTTCCTTCT				
GsSwobF	Forward	ATGAACTGCTTTTTCTTCCATCTTT	*ITS1*	532	*S. wobeseri*	[51]
GsSwobR2	Reverse	CTCCTCTTGAAGGTGGTCGTGT				
GsSglajamF1	Forward	TTTCGTAGCGCTGAGGAGATT	*ITS1*	~560	*S. glareoli*/*S. jamaicensis*	PS
GsSglajamR1	Reverse	TGCTTTTCTTCCTTTACTTTTGAATG				PS
Sgrau281	Forward	GCGGAGGAAAAGAAAATAACAAT	*28S* rRNA	~900	*Sarcocystis* spp. from rodents	[36]
Sgrau282	Reverse	CTATCGCTTAGGACCGGCTA				
GsSglaF1	Forward	GCAAAATGTGTGGTAAGTTTCACAT	*28S* rRNA	565	*S. glareoli*	[12]
GsSglaR1	Reverse	CCCTCTAAAAAGATGTTACCCTTCT				
GsSmicF1	Forward	TGTGGTAAGTTTCACATAAGGCTAA	*28S* rRNA	553	*S. microti*	[12]
GsSmicR1	Reverse	CTTTCTAAAAAGATGTACCTTCTCCT				
SgraupaukF	Forward	CGTATTTGCCCTGTGTCCTT	*28S* rRNA	~660	*Sarcocystis* spp. from rodents	PS
SgraupaukR	Reverse	GTCGTAGGTGCAAAGCATAACATC				PS

Ref.: reference; PS: present study; *: partial *18S* rRNA, complete *ITS1*, and partial *5.8S* rRNA.

**Table 2:** Genetic identification of avian *Sarcocystis* spp. in intestines of Common Buzzard based on *ITS1*.

Species	GenBank Accession Numbers	Intraspecific Similarity (%) *	Interspecific Similarity, Comparing with Most Closely Related Species (%) *
*S. cornixi*	PP937501	98.6–100	*S. kutkienae* 88.7–90.5
*S. halieti*	PP937502–PP937512	96.3–100	*S. columbae* 91.1–92.5
*S. kutkienae*	PP937513, PP937514	99.0–100	*S. cornixi* 88.4–89.1
*S. turdusi*	PP937515	98.4–100	*S. wobeseri* 84.8–86.1
*S. wobeseri*	PP937516–PP937522	99.0–100	*S. calchasi* 91.9–92.7

* All sequences available in GenBank are included in this comparison.

**Table 3:** Genetic identification of *Sarcocystis* spp. in intestines of Common Buzzard using rodents as their IHs.

Species	Genetic Region	Primer pairs	GenBank Acc No.	Percentage Intraspecific Similarity (Number of Sequences Determined in the Present Study) *	Percentage Interspecific Similarity, Comparing with Most Closely Related Species *
*S. glareoli*	*28S* rRNA	GsSglaF1/ GsSglaR1 and SgraupaukF/ SgraupaukR	PP938236– PP938265	99.8–100 (30)	*S. jamaicensis* 99.6–99.7
*S. glareoli*	*ITS1*	GsSglaF1/ GsSglaR1	PP937483–P937498	100 (16)	*S*. sp. Rod3 98.5
*S*. sp. Rod3	*28S* rRNA	GsSglaF1/ GsSglaR1 and SgraupaukF/ SgraupaukR	PP938266– PP938268	100 (3)	*S. jamaicensis* 99.8
*S*. sp. Rod3	*ITS1*	GsSglaF1/ GsSglaR1	PP937499– PP937500	100 (2)	*S. glareoli* 98.5
*S*. sp. Rod4	*28S* rRNA	SgraupaukF/ SgraupaukR	PP938269, PP938270	100 (2)	*S*. sp. Rod5 97.7
*S*. sp. Rod5	*28S* rRNA	SgraupaukF/ SgraupaukR	PP938271	not applicable (1)	*S.* cf. *strixi* and *S*. sp. Rod4 97.7

* All sequences available in GenBank are included in this comparison.

## Data Availability

The 28S *rRNA* and *ITS1* sequences of *Sarcocystis* species isolated form from intestinal mucosa scraping samples of the Common Buzzard from Lithuania are available in the GenBank database under accession numbers PP937483–PP937522 and PP938236–PP938271. Furthermore, the *ITS1* region sequence of *S. glareoli* from the bank vole containing partial *18S* rRNA, complete *ITS1*, and partial *5.8S* rRNA was submitted to GenBank with accession number PP937189.

## Supplementary Materials

The following supporting information can be downloaded at: https://www.mdpi.com/article/10.3390/ani14162391/s1, Table S1: Genetic identification of *Sarcocystis* species producing cysts in muscles or brains of rodents, in intestines of Common Buzzard by *28S* rRNA and *ITS1* sequences; Table S2: Percentage genetic similarity of four *Sarcocystis* species identified in the current study, and characterized by suggested small rodents—birds life cycle, based on *28S* rRNA and *ITS1* sequences.
