# Supplementary material for: The Genetic Identification of Numerous Apicomplexan Sarcocystis Species in Intestines of Common Buzzard (Buteo buteo)
Source: Animals (Basel). 2024 Aug 18;14(16):2391. doi: 10.3390/ani14162391 (PMC11350845; doi:10.3390/ani14162391)
Supplement: Supplementary file 1 [file animals-14-02391-s001.zip › animals-3144175-supplementary.pdf]

Table S1. Genetic identification of *Sarcocystis* species producing cysts in muscles or brains of rodents, in intestines of Common Buzzards by *28S* rRNA and *ITS1* sequences.

Isolate of Common Buzzard	Primer pair		
	**SgraupaukF/ SgraupaukR**	**GsSglaF1/GsSglaR1**	**GsSglajamF1/GsSglajamR1**
BbLT11	*Sarcocystis* sp. Rod4	*S. glareoli*	*S. glareoli*
BbLT13	*S. glareoli*	*S. glareoli*	*S. glareoli*
BbLT15	*S. glareoli*	*S. glareoli*	*S. glareoli*
BbLT16	*S. glareoli*	*S. glareoli*	*S. glareoli*
BbLT17	*S. glareoli*	*S. glareoli*	*S. glareoli*
BbLT18	*Sarcocystis* sp. Rod4	*Sarcocystis* sp. Rod3	*Sarcocystis* sp. Rod3
BbLT20	*S. glareoli*	*S. glareoli*	*S. glareoli*
BbLT21	*S. glareoli*	*S. glareoli*	*S. glareoli*
BbLT22	*S. glareoli*	*S. glareoli*	*S. glareoli*
BbLT24	*S. glareoli*	*S. glareoli*	*S. glareoli*
BbLT25	*S. glareoli*	*S. glareoli*	*S. glareoli*
BbLT28	*S. glareoli*	*S. glareoli*	*S. glareoli*
BbLT29	*S. glareoli*	*S. glareoli*	*S. glareoli*
BbLT33	*Sarcocystis* sp. Rod5		
BbLT34	*S. glareoli*	*S. glareoli*	*S. glareoli*
BbLT35	*S. glareoli*	*S. glareoli*	*S. glareoli*
BbLT36	*Sarcocystis* sp. Rod3	*Sarcocystis* sp. Rod3	*Sarcocystis* sp. Rod3
BbLT39	*S. glareoli*	*S. glareoli*	*S. glareoli*
BbLT40		*S. glareoli*	*S. glareoli*

Table S2. Percentage genetic similarity of four *Sarcocystis* species identified in the current study, characterized by suggested rodents' and birds' life cycles, based on *28S* rRNA and *ITS1* sequences. Genetic similarity values were included if query coverage was not less than 90%. Genetic similarity was calculated including sequences obtained in the current work and all available sequences of most closely related *Sarcocystis* species available in GenBank.

**Species**	**Primer pair**	***S. glareoli***	***S. microti***	***S. jamaicensis***	***S. strixi***	***S.* cf. *strixi***	***S. funereus***	***S.* sp. Rod2**	***S.* sp. Rod3**	***S.* sp. Rod4**	***S.* sp. Rod5**
S. glareoli	GsSglaF1/ GsSglaR1*	99.8–100	99.0	99.6	92.8	93.1	94.0	96.3	99.6	94.6	93.3
S. glareoli	SgraupaukF/ SgraupaukR*	99.8–100	98.7	99.7	93.6	93.8	94.2	96.6	99.3	95.0	94.0
S. glareoli	GsSglajamF1/ GsSglajamR1**	100	N/A	88.6	N/A	N/A	N/A	N/A	98.5	N/A	N/A
S. sp. Rod3	GsSglaF1/ GsSglaR1*	99.4–99.6	99.4	99.8	92.6	93.1	93.8	96.3	100	94.6	93.3
S. sp. Rod3	SgraupaukF/ SgraupaukR*	99.7	98.9	99.8	93.4	93.9	94.3	96.4	100	95.2	94.0
S. sp. Rod3	GsSglajamF1/ GsSglajamR1**	98.5	N/A	87.6	N/A	N/A	N/A	N/A	100	N/A	N/A
S. sp. Rod4	SgraupaukF/ SgraupaukR*	95.0	94.2	95.0	96.2	96.6	96.5	94.8	95.0	100	97.7
S. sp. Rod5	SgraupaukF/ SgraupaukR*	94.0	93.2	94.0	97.4	97.7	95.5–95.8	94.2	94.0	97.7	100

Grey shadows highlight higher genetic similarity values estimated for each *Sarcocystis* species analysed. N/A—not applicable, *—based on *28S* rRNA, **—based on *ITS1*.
